# Poisoning Pyridoxal 5-Phosphate-Dependent Enzymes: A New Strategy to Target the Malaria Parasite *Plasmodium falciparum*

**DOI:** 10.1371/journal.pone.0004406

**Published:** 2009-02-06

**Authors:** Ingrid B. Müller, Fang Wu, Bärbel Bergmann, Julia Knöckel, Rolf D. Walter, Heinz Gehring, Carsten Wrenger

**Affiliations:** 1 Department of Biochemistry, Bernhard Nocht Institute for Tropical Medicine, Hamburg, Germany; 2 Department of Biochemistry, University of Zürich, Zürich, Switzerland; University of Florida, United States of America

## Abstract

The human malaria parasite *Plasmodium falciparum* is able to synthesize *de novo* pyridoxal 5-phosphate (PLP), a crucial cofactor, during erythrocytic schizogony. However, the parasite possesses additionally a pyridoxine/pyridoxal kinase (PdxK) to activate B6 vitamers salvaged from the host. We describe a strategy whereby synthetic pyridoxyl-amino acid adducts are channelled into the parasite. Trapped upon phosphorylation by the plasmodial PdxK, these compounds block PLP-dependent enzymes and thus impair the growth of *P. falciparum*. The novel compound PT3, a cyclic pyridoxyl-tryptophan methyl ester, inhibited the proliferation of *Plasmodium* very efficiently (IC_50_-value of 14 µM) without harming human cells. The non-cyclic pyridoxyl-tryptophan methyl ester PT5 and the pyridoxyl-histidine methyl ester PHME were at least one order of magnitude less effective or completely ineffective in the case of the latter. Modeling *in silico* indicates that the phosphorylated forms of PT3 and PT5 fit well into the PLP-binding site of plasmodial ornithine decarboxylase (*Pf*ODC), the key enzyme of polyamine synthesis, consistent with the ability to abolish ODC activity *in vitro*. Furthermore, the antiplasmodial effect of PT3 is directly linked to the capability of *Plasmodium* to trap this pyridoxyl analog, as shown by an increased sensitivity of parasites overexpressing *Pf*PdxK in their cytosol, as visualized by GFP fluorescence.

## Introduction

Malaria is one of the most threatening diseases in the world, with more than 300 million infected people and up to 2 million fatalities annually, mainly young children and pregnant women living in Africa. The causative agent of the most severe form, tropical malaria, is *Plasmodium falciparum,* a protozoan parasite that sequesters in the red blood cells of its human host. As yet, there are no vaccines available and the few antimalarials are losing their efficacy due to the rapid spread of drug-resistant parasites. There is therefore an urgent need to find new strategies to interfere with the parasite's metabolism.

Pyridoxal 5-phosphate (PLP) is the active form of vitamin B6, which comprises the vitamers pyridoxine, pyridoxal and pyridoxamine as well as their related phosphate esters. It is a crucial cofactor for various enzymatic reactions, predominantly involved in the transformation of amino acids [1]. Many of the PLP-dependent enzymes are essential and therein offer the possibility to target diseases [2]. There are already promising attempts, such as the inhibition of γ-aminobutyric acid (GABA) aminotransferase by vigabatrin in epilepsy therapy [3] or of alanine racemase by antibacterial agents [4], [5]. The PLP-dependent enzyme ornithine decarboxylase (ODC) is a target in the fight against African trypanosomes, the causative agent of sleeping sickness, and against cancer [2], [6], [7]. ODC is the key enzyme in the formation of polyamines, which are essential for cellular proliferation and differentiation [8], [9], and accordingly, polyamine biosynthesis in the malaria parasite is also considered to be a possible drug target [10].

The reactions of PLP-dependent enzymes are highly diverse; however the initial formation of a Schiff base of PLP with the amino group of an amino acid is a common intermediate to all of them. Reduction of the Schiff base leads to a covalent coenzyme-substrate adduct resembling a transition-state analog with high affinity to the apoprotein [11]. Over thirty years ago, Heller et al. [12] synthesized several phosphopyridoxyl-amino acids that mimic the Schiff base intermediate involved in enzymatic decarboxylation. Among these, N-(5-phosphopyridoxyl)-ornithine inhibited rat liver ODC by binding to the holoenzyme in a non-competitive manner with respect to substrate and cofactor. Recently, a new analog, the BOC-protected pyridoxyl-ornithine conjugate (POB), was shown to inhibit human ODC in cells as well as the growth of various tumor and transformed cells effectively, whereas non-tumorigenic cells were less affected [13]. Another analog, pyridoxyl-histidine methyl ester (PHME), was able to inhibit human histidine decarboxylase in human mastocytoma cells without affecting proliferation [14].

Plants, bacteria, as well as some fungi are able to synthesize PLP *de novo*, whereas mammals entirely depend on the dietary uptake of pyridoxal, pyridoxine and pyridoxamine. They are converted into PLP via phosphorylation by pyridoxal/pyridoxine kinase (PdxK; EC 2.7.1.35) and subsequent oxidation by pyridoxine/pyridoxamine oxidase (EC 1.4.3.5). Although PdxK is widely distributed in human tissues, oxidase activity is low or absent in most cells [15]. Human erythrocytes have both enzymes and are proposed to be the main carrier of vitamin B6. PLP in red blood cells is mostly bound to proteins. To provide tissues with pyridoxal, PLP is dephosphorylated by vitamin B6 specific phosphatase within the erythrocyte [16]. The plasmodial parasite generates vitamin B6 *de novo* in its erythrocytic stage [17], [18]. The assembly of the plasmodial PLP-synthesis machinery follows a hierarchically defined course, which seems to be essential for activity [19]. In addition, the parasite possesses a functional PdxK [17], which in other protozoan parasites that lack *de novo* synthesis, such as trypanosomes, is the only enzyme activating salvaged pyridoxal [20].

In this report we present a new approach to target the malaria parasite by designing and testing novel pyridoxyl-adducts as antimalarials. In a first step these compounds are trapped upon phosphorylation in the parasite. In this state they mimic PLP and together with the substrate moiety they bind and inhibit efficiently PLP-dependent enzymes, which eventually kills the parasite. We validate their potency as substrates of *Pf*PdxK as well as their inhibitory effect on the PLP-dependent enzyme *Pf*ODC, and finally we demonstrate that the antiplasmodial effect can be directly linked to the activity of the plasmodial PdxK as transgenic parasites overexpressing *Pf*PdxK exhibit an increased sensitivity towards the pyridoxyl-analog.

## Results

### Pyridoxyl-amino acids are substrates of the plasmodial PdxK

**Figure 1:**
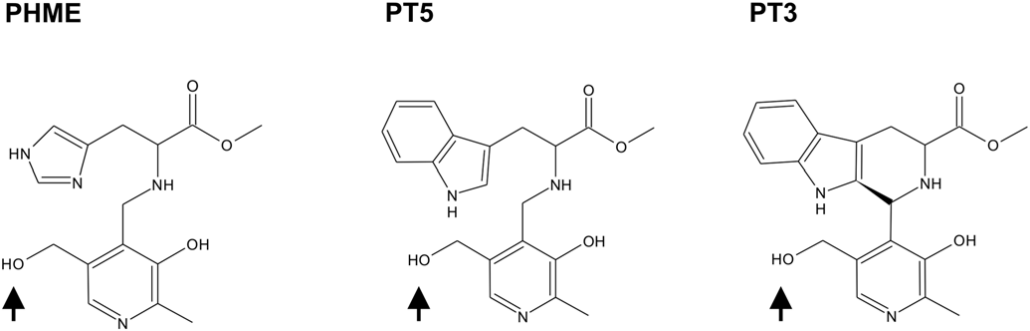
Structures of the pyridoxyl-amino acid adducts PHME, PT5 and PT3. The phosphorylation sites are indicated by arrows.

Our strategy is based on the fact that pyridoxal is phosphorylated by the plasmodial PdxK and the resulting PLP is thereby trapped within the parasite. In this respect the non-phosphorylated pyridoxyl-adducts PT3, PT5 and PHME (shown in Figure 1; their synthesis and that of their phosphorylated forms is described in the section Supporting information, Text S1) were tested as potential substrates of *Pf*PdxK. All three pyridoxyl-adducts were phosphorylated by the plasmodial enzyme with specific activities of 38, 20 and 64 nmol min^−1^ mg^−1^, respectively (Table 1).

**Table 1:** PT3, PT5, PHME and their phosphate esters as substrates of *Pf*PdxK or inhibitors of *Pf*ODC

Compounds	Specific activity of *Pf*PdxK (nmol min^−1^ mg^−1^) a)	Inhibition of *Pf*ODC activity IC_50_-value [µM] a)
PHME	64±4	n.d.
PT5	20±2	n.d.
PT3	38±4	n.d.
PPHME	n.d.	58±11
PPT5	n.d.	64±13
Pyridoxine b)	112±9	
Pyridoxal b)	50±4	
Pyridoxamine b)	30±2	

a) All results represent the mean values±standard deviations of three to six independent experiments; n. d. = not detectable.

b) Values are taken from [17].

### Phosphorylated pyridoxyl-amino acids inhibit *Pf*ODC in vitro

Phosphorylation of pyridoxal is required in order to obtain the active cofactor for the enzymatic reaction of PLP-dependent enzymes. The activity of ODC depends on PLP and its competition by the pyridoxyl-adducts was measured as a representative for PLP-dependent enzymes. The non-phosphorylated pyridoxyl-adducts, PHME, PT5 and PT3, did not inhibit the plasmodial ODC activity in the standard *in vitro* assay (Table 1). Only the phosphopyridoxyl-adducts PPHME and PPT5 act as inhibitors on the plasmodial ODC with IC_50_-values of 58 µM and 64 µM, respectively, as shown in Table 1. Since PPT3 was not available its inhibitory potential was analyzed by *in silico* modeling.

### Structural analysis of *Pf*ODC binding to PPT3 and PPT5

**Figure 2:**
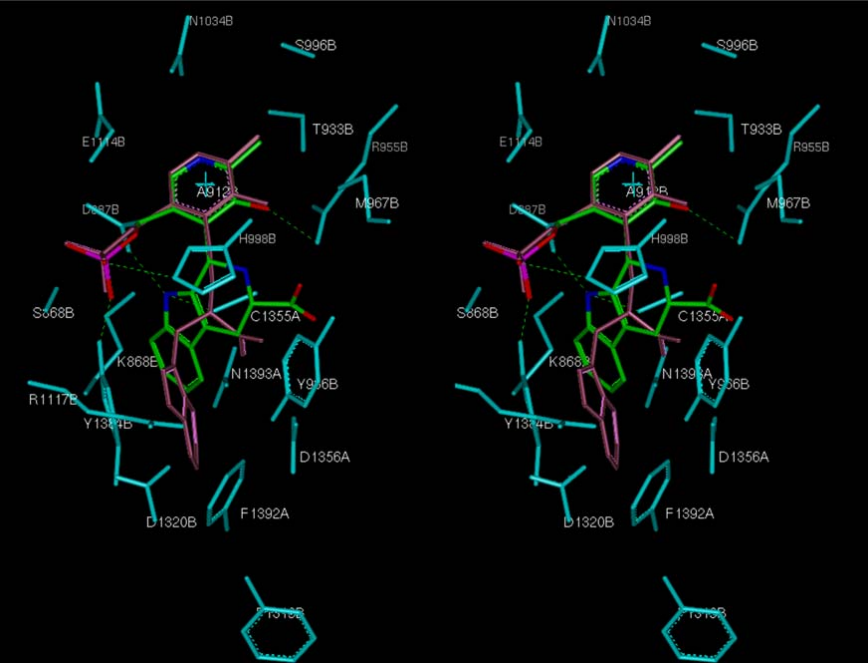
Stereo view of the putative binding mode of PPT3 and PPT5 in the active site of *Pf*ODC. Residues surrounding the coenzyme-substrate analogs within a distance of 3 Å are indicated in cyan. PPT5 is colored in pink and the cyclic form PPT3 is colored in green and by default atom types (O, N). The hydrogen bonds formed by PPT3 and *Pf*ODC are shown with green dotted lines.

The active species of PT3 and PT5 in cells are presumed to be their phosphorylated forms. To study the binding mode in the active site of plasmodial ODC and to elucidate binding differences, PPT3 and PPT5 were modeled into the active site of *Pf*ODC (Figure 2). The 5-phosphate-pyridoxyl motif forms hydrogen bonds with Ser868B, Met967B, His998B and Tyr1384B in both compounds. The side chains of both PPT3 and PPT5 occupy the pocket formed by Asp887B, Lys868B, Cys1355A, Asn1393A, Arg1117B, Asp1320B, Asp1356A and the hydrophobic residues Tyr1384B, Tyr966B, Phe1392A. The indole ring of PPT3 and PPT5 is engaged in hydrophobic interactions with Tyr1384B. The N atom of the indole ring of the cyclic PPT3 can form hydrogen bonds with Asp887B and Cys1355A, whereas the non-cyclic PPT5 can form one with Asp1320B.

### PT3 exhibits distinct antiplasmodial activity

**Figure 3:**
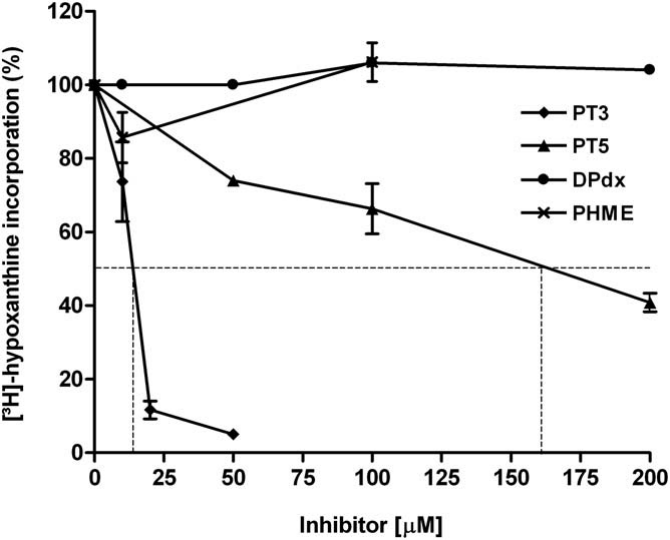
Effect of PT3, PT5 and PHME on cultured *Plasmodium.* Proliferation was determined after 48 h incubation by the [^3^H]-hypoxanthine incorporation assay described in Material and Methods. The experiment presented is representative of at least three independent analyses each in triplicate.

To analyze the potential of the pyridoxyl-amino acid conjugates in blocking the proliferation of cultured *P. falciparum*, the parasites were treated with the non-phosphorylated forms. Additionally, deoxypyridoxine, known to be effective against *P. lophorae* [21], was tested as a control. While deoxypyridoxine and PHME had no effect on parasite growth, PT3 inhibited the proliferation of cultured *Plasmodium* with an IC_50_-value of 14 µM (Figure 3). PT5 also showed antiplasmodial activity, however, the apparent IC_50_-value was more than one order of magnitude higher. The phosphorylated compounds did not inhibit plasmodial growth at all (data not shown). Interestingly, the effect of PT3 on the growth of mammalian cells was marginal. At a concentration of 50 µM PT3 the growth was decreased to about 75% of the untreated control. Treating the cells with 100 µM PT3 only slightly increased inhibition further (Table 2).

**Table 2:** Effect of PT3 on mammalian cell lines

Mammalian cell lines	Growth inhibition (%)*	
	50 µM PT3	100 µM PT3
RD	74±5	60±7
A 549	73±8	65±8
FRHK 4	76±6	73±9

* Of untreated control; all results represent the mean values±standard errors of at least three independent experiments.

### Localization of the *Pf*PdxK

**Figure 4:**
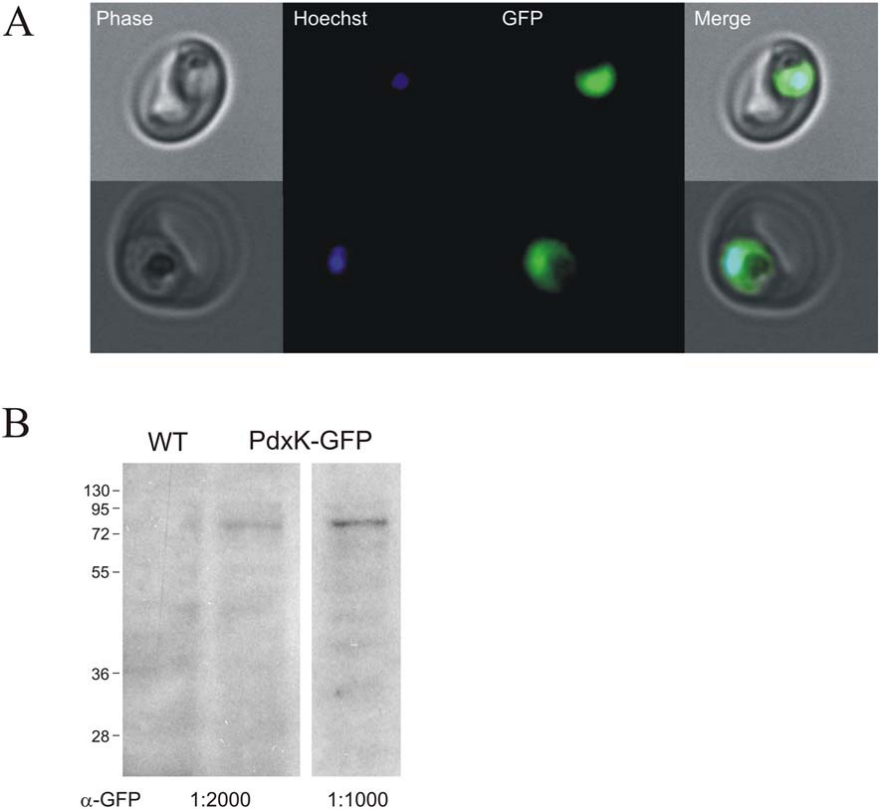
Localization of *Pf*PdxK-GFP in living parasites by fluorescence microscopy. A) Fluorescence of the GFP moiety (green) is found in the parasite's cytosol. The nucleus is stained with Hoechst dye (blue). B) Total protein homogenates of WT and *Pf*PdxK-GFP parasites were separated by SDS-PAGE and *Pf*PdxK-GFP was visualized by western blot analysis using an anti-GFP antibody diluted 1∶2000 (left panel) and 1∶1000 (right panel).

In order to determine the localization of the plasmodial PdxK, the open reading frame of *gfp* was fused to the 3′-end of *pfpdxk* resulting in a *Pf*PdxK-GFP chimera. Three weeks post-transfection, cells resistant to the selection drug WR99210 were obtained and successful transfection of the construct was confirmed by plasmid rescue (data not shown). The *Pf*PdxK-GFP chimera revealed an even distribution in the parasite's cytosol (Fig. 4A) and its expression was confirmed by western blot analysis showing a band of the expected size of 82 kDa only in the transgenic cell line (Fig. 4B).

### *P. falciparum* overexpressing *Pf*PdxK is more sensitive towards PT3

**Figure 5:**
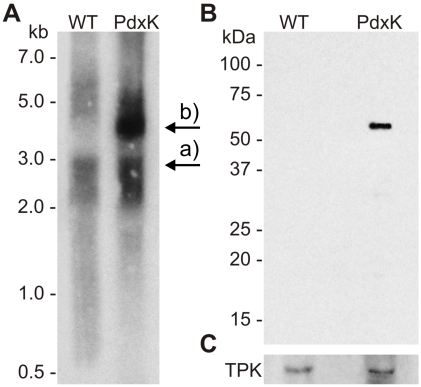
Analysis of *Pf*PdxK-strep expression in *P. falciparum*. A) RNA isolated from wild-type (WT) and transgenic *Pf*PdxK-strep (PdxK) cells was analyzed by a northern blot, on which beside the endogenous *pdxk* (a) an additional band of approx. 4 kb was detected only in PdxK, corresponding to the expected size of the exogenous transcript (b). B) Total protein homogenates of WT and PdxK parasites were separated by SDS-PAGE and the expression of *Pf*PdxK-strep was visualized by western blot analysis using an anti-strep antibody. C) The western blot was reprobed with a specific anti-thiamine pyrophosphokinase (TPK) antibody as loading control.

**Figure 6:**
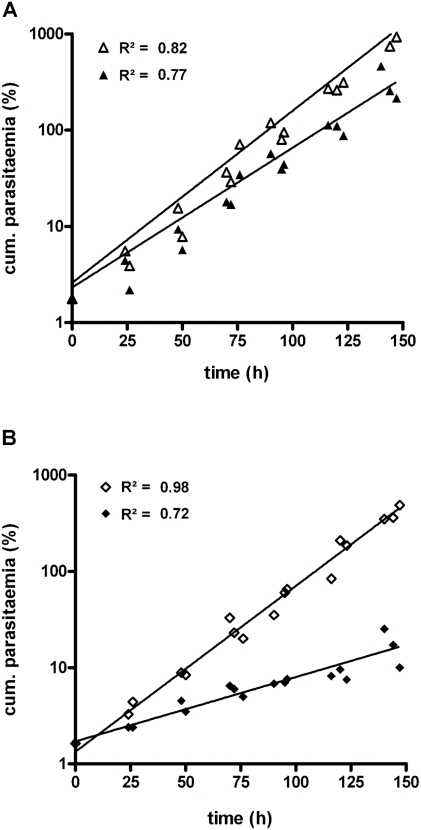
Effect of PT3 on the growth of transgenic *P. falciparum*. A) Wild-type as control (triangle) and B) *Pf*PdxK-strep overexpressing cells (diamond) were cultured with (filled) and without (open) 7 µM PT3 for 150 h. Cumulative parasitaemias were calculated by extrapolation from observed parasitaemias and the corresponding dilution factors that were employed at each sub-culturing step. The data consist of three independent monitorings plotted on a semi-logarithmic scale with exponential regression shown as trend lines with respective R^2^-values.

Since *Pf*PdxK presumably activates the prodrug PT3, thereby triggering inhibition of parasite growth, another transgenic *P. falciparum* cell line was created. This cell line overexpressed *Pf*PdxK fused to a C-terminal strep tag II encompassing ten amino acid residues (*Pf*PdxK-strep), thus avoiding possible steric influences of the GFP protein. The expression of *Pf*PdxK-strep was verified by northern blot analysis of total RNA (Figure 5A). Additional to the endogenous mRNA of about 3 kb, present in wild-type and *Pf*PdxK-strep parasites (a), a 4 kb band occurred only in the transgenic *Pf*PdxK-strep expressing cell sample (b). The corresponding *Pf*PdxK-strep protein was further verified by western blot analysis, using a monoclonal antibody against the fused strep tag (Figure 5B). To analyze the effect of PT3 on the transgenic *Pf*PdxK-overexpressing cells as well as on wild-type parasites, proliferation was monitored over a period of 6 days. The experiment clearly revealed that *Pf*PdxK-overexpressing cells that were treated with 7 µM PT3, corresponding to half the IC_50_-value, exhibited a considerably increased doubling time (45.0 h, calculated as described in Materials and Methods), in comparison to untreated *Pf*PdxK-strep cells (17.5 h). This parameter was only slightly altered for wild-type cells from 16.9 h for the untreated control to 20.7 h for the culture treated with PT3 (Figure 6).

## Discussion

The general lack of progress in malaria control depends not least on account of resistance to common antimalarials [22]. In this respect, not only the search for novel drug targets in the parasite's metabolism but also new approaches should be considered to fight the parasite.

We propose here a strategy to poison PLP-dependent enzymes in order to kill the human malaria parasite *P. falciparum*. PLP is a cofactor in more than 140 distinct enzymatic reactions, corresponding to approx. 4% of all classified activities [1]. Many of the PLP-dependent enzymes are essential; once blocked, the death of the parasite follows, as reported for the plasmodial ornithine decarboxylase (*Pf*ODC) [10], [23]. The idea behind the proposed strategy is hence to target critical PLP-dependent enzymes of the parasite simultaneously by using pyridoxyl-adducts as prodrugs.

Wu et al. [14] showed that the pyridoxyl-histidine methyl ester (PHME) acts as a potent transition-state inhibitor of histidine decarboxylase (hHDC) in human cells. When PHME was applied to cultured *P. falciparum* no significant growth inhibition was observed; similar to the result obtained with the pyridoxine analog deoxypyridoxine, although the latter was previously reported to be an effective inhibitor in a different system [21]. Either these two compounds do not interfere sufficiently with the parasite's metabolism or have only a limited uptake. Since the erythrocyte, the host cell that harbors *Plasmodium*, is regarded as the main carrier of vitamin B6 in the human [15], the question arises whether the strategy of poisoning PLP-dependent enzymes in the parasite is at all applicable. However, it was shown that PLP is immediately bound by hemoglobin [24] and hence of limited availability for the parasite. It therefore remains to be clarified to what extent the parasite relies on salvage, given that the *de novo* synthesis of PLP seems to be functional in the blood stage, during which *Plasmodium* can survive without additional pyridoxine [25].

In this study we present the newly synthesized pyridoxyl-tryptophan methyl esters PT3 and PT5 (Figure 1). The cyclic PT3 exhibits an antimalarial effect in the low micromolar range, with an IC_50_-value of 14 µM, whereas the non-cyclic PT5 is one order of magnitude less effective (Figure 3). PT3 treatment of human cells revealed only a moderate effect (as shown in Table 2), suggesting that the impact of PT3 is parasite-specific. Moreover, effective inhibition of *Plasmodium* at the cellular level with this pyridoxyl-analog demonstrated that *de novo* synthesis of PLP is unable to compensate for the adverse effect of PT3 on parasite survival. While we assume that, *in vivo*, there is a dual provision to the parasite's PLP pool, further experiments concerning uptake and functional analyses on knock-out parasites would have to be performed to establish the contributions and relative importance of salvage and *de novo* synthesis.

As well as demonstrating their antimalarial effect the studies presented here show that both compounds, PT3 and PT5, are phosphorylated by the *Pf*PdxK *in vitro* at levels comparable to those for the natural substrates pyridoxal and pyridoxamine (Table 1). From this we assume that the pyridoxyl-analogs are likely to be trapped in the parasite. Their trapping in the erythrocyte can be discounted since, beside a PdxK, the host also possesses pyridoxal phosphatase activities [16].

In order to examine whether the phosphorylated pyridoxyl-amino acid adducts are able to interact with their assumed target enzyme, the inhibitory effect on the plasmodial ODC activity was measured. As expected, only the phosphorylated analogs interfered with the decarboxylation of ornithine to putrescine and were thus able to displace PLP in *Pf*ODC (Table 1). The fact that pyridoxyl-histidine and -tryptophan inhibit ODC is contrary to the generally accepted view that such transition state inhibitors should be designed only as coenzyme-substrate adducts.

As PPT3 was not available, its inhibitory action on plasmodial ODC could not be experimentally measured and thus its interaction was studied *in silico*. Molecular modeling of PPT3 and PPT5 in the active site of *Pf*ODC predicted that these would have different binding affinities to the enzyme. PPT3 forms hydrogen bonds with Asp887B and Cys1355A of *Pf*ODC, whilst PPT5 can form only one, with Asp1320 (Figure 2). The concerted hydrogen bonding of the N atom of the indole ring of PT3 with Asp887B and Cys1355A found in the modelled complex, which results in an additional hydrogen bond in comparison to the complex with PPT5, could account for the better binding of PPT3. Whether this is the main contribution or whether additional energetically favourable interactions are present is beyond the scope of the present modelling studies. Notably, Cys1355A has been assumed to be the catalytic residue in *Pf*ODC [26], with a function comparable to Cys360 in mammalian [27] and trypanosomal ODC [28]. Cys360 modification totally abolishes ODC activity, as demonstrated with the well-known suicide inhibitor DFMO [27].

The use of pyridoxyl-adducts as competitive transition-state inhibitors has already been tried by many groups and their potency confirmed [12], [29]. However, using such compounds as prodrugs against *Plasmodium* had not been tested so far. The strategy proposed here is highly dependent on the phosphorylation by *Pf*PdxK to activate and trap the phosphorylated prodrug in the cell. The key role of *Pf*PdxK could be demonstrated by overexpressing a transgenic copy of *Pf*PdxK in the parasite. By means of a GFP chimera, a cytosolic localization of *Pf*PdxK - important for trapping PT3 - could be observed (Figure 4). As GFP might disturb the enzyme activity of PdxK, a construct carrying a small strep tag (10 amino acids) instead of GFP (238 amino acids) was also generated. Expression of the *Pf*PdxK-strep fusion product, previously shown to maintain PdxK activity [17], was subsequently confirmed by western and northern blot analyses (Figure 5). Since in this case *Pf*PdxK is not the target enzyme but the activator of the prodrug PT3, an increased sensitivity of transgenic *Pf*PdxK overexpressing cells would be expected relative to wild-type. Consistent with the proposed strategy, monitoring parasite growth over several days clearly revealed that the impact of PT3 on *Pf*PdxK overexpressing cells was considerably stronger. The growth rate of these cells was reduced by 61% relative to the untreated control (Figure 6B), whereas the proliferation of the wild-type was only inhibited by 19% (Figure 6B). From that we conclude that a time-dependent accumulation of PPT3 in the parasite's cytosol occurred, emphasizing the key role of *Pf*PdxK.

**Figure 7:**
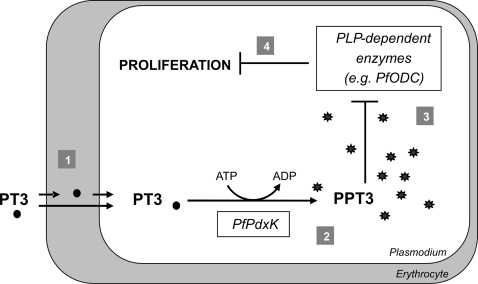
Schematic action of PT3. 1) Uptake of PT3 occurs either via a parasite specific transport system directly into the parasite or indirectly via the host cytosol where PT3 might be phosphorylated and dephosphorylated by the human enzymes, PdxK and pyridoxal phosphatase, respectively. 2) Activation of PT3 upon phosphorylation by *Pf*PdxK leads to an accumulation of the compound as PPT3 in the cell. 3) PPT3 competes with PLP for binding in the active site of *Pf*ODC or other PLP-dependent enzymes. 4) Inhibition of crucial PLP-dependent enzymes, such as ODC, results in growth arrest.

Our results suggest that the pyridoxyl-tryptophan derivative enters the parasite, possibly directly via an amino acid transporter. After phosphorylation, PPT3 displaces PLP from the active site of ODC (and other PLP-dependent enzymes) or binds directly to newly synthesized apoenzymes which ultimately arrests growth of *P. falciparum* (Figure 7). The significant superiority of the cyclic form (PT3) compared to the open form (PT5) of pyridoxyl-tryptophan in inhibiting proliferation of the parasite can be explained by differences in binding of *Pf*ODC as indicated by the molecular modeling studies.

The selective inhibition profile for proliferation achieved with PT3 - IC_50_ of 14 µM for *P. falciparum* (Figure 3) and IC_50_>100 µM for human cells (Table 2) - suggests that the approach of poisoning *Plasmodium* with PLP-mimicking compounds holds considerable promise. Further studies will be required to show whether, besides ODC, additional plasmodial enzymes are effectively targeted and to improve the prodrugs by appropriate structural modifications.

## Materials and Methods

### Materials

Restriction enzymes and ligase were purchased from New England Biolabs, USA. Oligonucleotides were from Operon, Germany. Strep-Tactin-Sepharose, anhydrotetracycline and dethiobiotin were from IBA (Institut für Bioanalytik, Germany). Radioactive compounds: [U-^3^H]-hypoxanthine, [γ-^33^P]-ATP and [1-^14^C]-L-ornithine were from GE Healthcare, Germany. All other chemicals were from Sigma, Germany, unless otherwise stated.

### Enzyme assays

Recombinant expression using an anhydrotetracycline inducible system and affinity purification via strep tag of the plasmodial PdxK as well as the bifunctional *P. falciparum* AdoMetDC/ODC were carried out as described previously [30]. The enzyme assay of the *Pf*PdxK was performed in 70 mM potassium phosphate buffer, pH 6.5, containing either 200 µM pyridoxine according to Wrenger et al. [17] or up to 200 µM of the non-phosphorylated pyridoxyl-adducts (PHME, PT5 and PT3), 5 mM MgCl_2_, 500 µM (2 µCi ^33^P) ATP as well as 2.5 µg protein in a total volume of 100 µl. The reaction took place at 37°C for 1 h. The data were analyzed using GraphPad Prism 4.0. *Pf*ODC activity was assayed as described [26] by measuring the formation of ^14^CO_2_ from [^14^C]-L-ornithine at 37°C. The standard assay contained, in a final volume of 250 µl: 40 mM Tris-HCl buffer, pH 7.5, 1 mM dithiothreitol, 1 mM EDTA, 10 µM pyridoxal 5-phosphate, 100 µM (125 nCi ^14^C) L-ornithine and 2 µg recombinant *Pf*ODC/AdoMetDC. The reaction occurred at 37°C for 30 min. The inhibitory effect, expressed as the IC_50_-value, of the phosphopyridoxyl-adducts (PPHME and PPT5) was determined at concentrations of 50, 100 and 200 µM.

### Molecular modeling

The model structure of *Pf*ODC together with bound PLP and DFMO [31] was obtained from the protein data bank (http://www.rcsb.org, PDB code: 1M9V). The active site model of *Pf*ODC with PPT3 and PPT5 was generated on the basis of the known 3D coordinates of the complex of *Pf*ODC with PLP-DFMO followed by energy minimization with the InsightII/Built module (steepest descents, up to a maximum derivative of 20 kcal/mol, consistent valence force field, CVFF). The structures were further optimized by energy minimization using the InsightII/Discover module (conjugation gradient, until a maximum derivative of 0.01 kcal/mol was reached, CVFF) according to Wu et al. [14].

### Inhibition assays on cultured *P. falciparum* and mammalian cells

*P. falciparum* 3D7-strain was maintained in continuous culture according to Trager and Jensen as modified [23]. The parasites were grown in human erythrocytes (O+), RPMI 1640 medium supplemented with 25 mM HEPES, 20 mM sodium bicarbonate and 0.5% AlbuMAX II (Invitrogen, Germany) at 4% hematocrit. The cells were cultivated in 90 mm Petri dishes (Nunc, Denmark) and incubated at 37°C in presence of 90% N_2_, 5% O_2_ and 5% CO_2_. The impact of deoxypyridoxine and the pyridoxyl-adducts PHME, PT5 and PT3 on the erythrocytic stages of *P. falciparum* was determined by using the [^3^H]-hypoxanthine incorporation assay as described [23] and the IC_50_-value of PT3 was calculated from sigmoidal inhibition curves using GraphPad Prism 4.0. Additionally, 50 and 100 µM PT3 was tested against human cell lines RD and A 549 as well as the rhesus monkey cells FRHK 4 cultured in DMEM complete medium (PAA Laboratories, Austria) containing 7.5% FCS (Invitrogen) at 5% CO_2_ for 48 h at 37°C in 96 well plates. Cell viability was determined by incorporation of [^3^H]-hypoxanthine as described above using 10000 cells per well. To analyze the long term effect of PT3 on the PdxK-overexpressing and wild-type parasites, growth rates were monitored for several days by light microscopy of Giemsa-stained thin smears with a starting parasitaemia between 1 and 2%. Growth curves were generated with GraphPad Prism 4.0 using an exponential equation to calculate the slopes.

### Generating transgenic parasites

To overexpress the plasmodial PdxK in the parasite as a strep tag II (strep) fusion protein or as a *Pf*PdxK-GFP chimera, two constructs were generated carrying *pfpdxk* fused to C-terminal strep or GFP, respectively. The open reading frame of *pfpdxk* was amplified by PCR from gDNA using the Expand Long Template PCR System (Roche Molecular Biochemicals, Germany) and the specific oligonucleotides *Pf*PdxK-pARL-kpn-S (sense) 5′-GAGA**GGTACC**ATGAAGAAGGAAAATATTATCTCC-3′ and *Pf*PdxK-strep-avr-AS (antisense) 5′-GAGA**CCTAGG** CTATTTTTCGAACTGCGGGTGGCTCCAAGCGCTAAAAAAAACAGGCTCTTCTTTAATTAAAATATC-3′ for the strep (underlined) fusion protein and *Pf*PdxK-pARL-avr-AS (antisense) 5′-GAGA**CCTAGG**AAAAAAAACAGGCTCTTCTTTAATTAAAATATC-3′ for the *Pf*PdxK-GFP chimera to obtain a 1451 bp or 1484 bp fragment, respectively, comprising the full *pfpdxK* ORF without the stop codon and with strep, respectively. The fragments were cloned into pARL1a- [32] containing GFP with KpnI and Avr II restriction enzymes (bold) resulting in the constructs pARL-*Pf*PdxK-strep and pARL-*Pf*PdxK-GFP. Both constructs were confirmed by automated sequencing on an ABI 377 (Applied Biosystems Inc., Germany) and the plasmid DNA for transfection was obtained using a Maxi Prep Kit (Qiagen, Germany). Prior to transfection, parasites were synchronized with 5% sorbitol [33] and cultivated for at least one additional cycle of schizogony. Transfection was carried out by spontaneous uptake of DNA [34]. Before electroporation, 400 µl red blood cells were washed in cytomix [35] and mixed with 100 µg circular plasmid DNA dissolved in 30 µl Tris-EDTA. Electroporation was performed in a 0.2 cm gap cuvette (Biorad, Germany) at 310 V and 900 µF in an EasyJect Gene Pulser (EquiBio, Germany). Red blood cells pre-loaded with DNA in this way were transferred into prewarmed medium and provided with 50 µl red blood cells infected with 10% schizonts. The next day the culture medium was supplemented with 5 nM WR99210 (Jacobus Pharmaceutical Company, USA) as the selection drug. The *Pf*PdxK-strep parasites obtained were maintained in 50 nM WR99210 to induce overexpression of the *Pf*PdxK-strep fusion protein.

### Analysis of transgenic parasites

Wild-type and *Pf*PdxK-strep overexpressing parasites were separated from the host cell with saponin [36] and kept at −20°C for protein isolation or resuspended in Trizol (Invitrogen) for RNA extraction according to the manufacturer's instructions; 25 µg total RNA were analyzed by northern blotting [37] using a radiolabeled *pfpdxk*-probe. Total plasmodial protein extract was obtained by boiling the parasites at 95°C for 5 min in an SDS- and mercaptoethanol-containing buffer. Equal amounts (15 µg) were separated on 10% SDS-PAGE and subsequently transferred to a nitrocellulose membrane (Schleicher & Schüll, Germany). Expression of *Pf*PdxK-GFP and of *Pf*PdxK-strep was analyzed using either a polyclonal anti-GFP antibody (1∶1000 and 1∶2000; Invitrogen) or a monoclonal anti-strep antibody (1∶5000; IBA) and HRP-coupled secondary anti-rabbit or anti-mouse antibodies (1∶10000; Invitrogen, Germany), respectively. The blot was stripped and reprobed with a polyclonal antibody against plasmodial thiamine pyrophosphokinase (TPK) (1∶10000; [38]) and the secondary anti-rabbit HRP coupled antibody (1∶10000, Dianova, Germany). The detection was performed using ECL (Millipore, Germany). The green fluorescence of the *Pf*PdxK-GFP chimera was detected and captured in live cells using a Zeiss Axioskop 2 and the OpenLab software (Improvision, Germany). The parasite's nuclei were visualized with 0.2 µg/ml Hoechst 33342 dye (Invitrogen, Germany).

## Supporting Information

Text S1 (0.02 MB DOC)
