# Supplementary material for: Poisoning Pyridoxal 5-Phosphate-Dependent Enzymes: A New Strategy to Target the Malaria Parasite Plasmodium falciparum
Source: PLoS One. 2009 Feb 6;4(2):e4406. doi: 10.1371/journal.pone.0004406 (PMC2634962; doi:10.1371/journal.pone.0004406)
Supplement: Text S1 — (0.02 MB DOC) [file pone.0004406.s001.doc]

**Text S1: Synthesis of pyridoxyl-amino acids**

Non-phosphorylated and phosphorylated pyridoxyl-histidine (PHME and PPHME) and tryptophan (PT3, PT5 and PPT5) derivatives were synthesized as described [14]. Briefly, PL or PLP (1 mmol) and NaHCO_3_ (2 mmol) were dissolved in 5 ml ethanol, and the substrate analogs (1.05 mmol) together with NaHCO_3_ (3 mmol) in 10 ml ethanol. Both solutions were mixed at 0ºC and stirred at room temperature for 2 h. NaBH_4_ (125 mg) was then added in small portions to the solution on ice. After 30 min, acetic acid (100 %) was added to pH 5 to stop the reaction. The solvents were removed with a vacuum dryer; the residue was dissolved in water and subjected to FPLC or HPLC for analysis and purification. PHME and PPHME were analyzed and purified with FPLC equipped with a mono S HR5/5 column (Amersham Pharmacia Biotech, Sweden). The samples were loaded and separated with a flow rate of 0.5 ml/min using the following elution steps: 0-5 min, 100 % solvent A (50 mM acetic acid pH 4.6) and 0 % solvent B (50 mM acetic acid, pH 4.6, 1 M NaCl); 5-30 min, 0-100 % B; 30-40 min, 100 % B. Detection was at 290 nm. PT3, PT5 and PPT5 were analyzed and purified with a Kromasil C18 column (4.6 x 250 mm; Akzo Nobel, Sweden). The samples were loaded and separated with a flow rate of 1 ml/min using the following elution steps: 0-5 min, 100 % solvent A (3 % acetonitrile/0.1 % TFA) and 0 % solvent B (80 % acetonitrile/0.1 % TFA); 5-20 min, 0-100 % B; 20-30 min, 100 % B. A preparative Kromasil C18 column (50.8 x 250 mm; Akzo Nobel, Sweden) with a flow rate of 15 ml/min was used under preparative conditions: 0-36 min, 100 % solvent A (3 % acetonitrile/0.1 % TFA) and 0 % solvent B (80 % acetonitrile/0.1 % TFA); 36-92 min, 0-80 % B; 92-120 min, 80-100 % B, followed by 100 % B. Elution was recorded at 290 nm. The eluted compounds were collected as individual peaks from the C18 columns (analytical column and preparative column). Retention times were for PT5, 17.6 min (analytical column) and 84 min (preparative column); for PPT5 slightly less, for PT3 20.1 min (analytical column) and 90 min (preparative column). The purified compounds obtained (purity >95 %) were identified by ESI-MS and ESI-MS-MS methods. PHME: theoretical mass 320.15, measured mass [M+H]^+^ 321.15; PPHME: 400.11, 401.11; PT5: 369.17, 370.17; PPT5: 449.14, 449.91; PT3: 367.15, 367.97. PPT3 could not be synthesized in adequate amounts and purity.
